# Clinical leadership and coping strategies in times of COVID-19: observational study with health managers in Mendoza

**DOI:** 10.1186/s12913-023-09792-y

**Published:** 2023-08-16

**Authors:** Eva Gil-Hernández, Andrea Falaschi, Irene Carrillo, Viviana Rodríguez, María Belén Peralta Roca, Ezequiel García-Elorrio, José Joaquín Mira

**Affiliations:** 1 Atenea Research Group, Foundation for the Promotion of Health and Biomedical Research of Valencia Region (Fisabio), Sant Joan d’Alacant, Spain; 2 Ministry of Health, Social Development and Sport, Government of Mendoza, Mendoza, Argentina; 3 Health Psychology Department, Miguel Hernández University, Avenida de la Universidad s/n, Elche, 03202 Spain; 4 Institute for Clinical Effectiveness and Health Policy, Buenos Aires, Argentina; 5 Alicante-Sant Joan d’Alacant Health District, Alicante, Spain

**Keywords:** COVID-19, Pandemic, Healthcare workers, Adaptation, Leadership

## Abstract

**Background:**

The outbreak of the COVID-19 pandemic required an immediate response to the healthcare challenges it posed. This study was conducted to identify actions that helped healthcare professionals to overcome the initial impact in Mendoza (Argentina).

**Methods:**

A cross-sectional study was carried out in a non-random sample of managers and staff of the public health system of Mendoza (Argentina) (n = 134). An ad-hoc and voluntary survey was carried out with 5 multi-response questions that combined questions referring to the management of the pandemic at the organizational level with others referring to coping at the individual level. The survey questions were formulated based on the results of six focus groups that were conducted previously. Descriptive frequency analysis was performed.

**Results:**

60 people agreed to participate and 45 answered the full questionnaire. At both the organizational and individual level, there was consensus with at least 50% of votes. The most outstanding at the organizational level was “Prioritize the need according to risk” and at the individual level it was “Support from family or friends”, being also the most voted option in the whole questionnaire.

**Conclusions:**

The responses that emerged for coping with COVID-19 must be seen as an opportunity to identify strategies that could be effective in addressing future crisis situations that jeopardize the system’s response capacity. Moreover, it is essential to retain both changes at the organizational level (e.g., new protocols, multidisciplinary work, shift restructuring, etc.) and coping strategies at the individual level (e.g., social support, leisure activities, etc.) that have proven positive outcomes.

## Background

The emergence of a novel coronavirus in December 2019 [1] required an immediate response to the healthcare challenges it entailed, particularly during the first wave. The impact on both the affected population and the professionals committed to their care was considerably more compared to other health emergency situations due to the scarcity of resources, contradictory and insufficient information and the global nature of the phenomenon [2].

Acute stress, compassion fatigue, and moral injury, along with psychosomatic symptoms, insomnia, mood swings, irritability, and frustration were common among healthcare personnel [3, 4]. The fear of contagion, including the risk of transmission to loved ones, and the significant number of infected cases among health professionals [5] threatened the response capacity of health institutions [6].

The first COVID-19 case in Argentina was recorded on March 3, 2020, and was linked to a passenger arriving from Milan, Italy. On March 20 of the same year, the nationwide quarantine was imposed [7]. The first wave of the pandemic spanned from March 23 to December 22, 2020, approximately [8]. During that time, there were 1,555,279 confirmed cases and 42,254 deaths, belonging 59,189 of the total cases to the province of Mendoza [9]. According to the Ministry of Health of the Government of Argentina report for that period, the positivity rate among healthcare workers was 64,958, equivalent to 1,197.9 cases per 10,000 health workers [10] and, specifically in Mendoza, the seroprevalence (determined by ECLIA, Elecsys® anti-SARS-CoV-2 test), in January 2021, was 33.57% (95% CI 31.02–36.22) [11].

The surge in COVID-19 infections resulted in the collapse of healthcare systems worldwide, exposing healthcare personnel to work overload and unprecedented mental stress. The exceptional circumstances they faced, such as the high number of deaths and social exclusion, created an ideal scenario for anxiety, burnout, and post-traumatic stress to manifest [12]. In these circumstances, there were some defections (in different numbers depending on the countries), but a majority of the professionals committed to their work and contributed to moving forward a good number of COVID-19 and non-COVID-19 patients in adverse circumstances. Continuing with the working days, returning every day to work being aware of the situation they were going to face, was a challenge both at a personal and organizational level. In this environment, improvised and other planned measures emerged to alleviate this situation.

Several studies have highlighted the toll the pandemic has taken on the mental health of healthcare workers, especially during the first waves. Del Pozo-Herce et al. [13] conducted a study in Spain in which they found that the pandemic had negatively impacted the stress and emotional well-being of healthcare workers, with young women being the most affected. In Argentina, several studies have pointed out burnout as one of the predominant syndromes associated with chronic stress among healthcare personnel during the pandemic [14, 15]. Burnout is a major concern as it directly affects patient care [16]. Healthcare professionals are a fundamental component of healthcare systems, and their well-being is essential to ensure quality in healthcare.

The purpose of this study was to identify valuable insight that can be applied in future crises exploring what changes made during the pandemic were positive for healthcare teams and what coping mechanisms were most frequently utilized by healthcare workers to navigate the challenges.

## Methods

Cross-sectional study in a non-random sample of managers and staff of the public health system of Mendoza (Argentina) carried out in March 2022. The Research Committee of the San Juan University Hospital in Alicante (April 8, 2020) approved the study protocol in accordance with the Declaration of Helsinki.

### Setting

At the time of the study, the population of the province of Mendoza was approximately two million inhabitants, with a total of 22 public hospitals, 272 health centers, 51 health posts, and 25 community integration centers, in which around 21,000 professionals of various professional profiles were working. Of these, approximately 15,000 were involved in providing care during the COVID-19 pandemic. In the province of Mendoza, the first case of COVID-19 was registered on March 21, 2020, with 166,545 cases identified and 4,667 deaths recorded from this cause until March 2022. After a decrease in the number of initial cases, two peaks of new cases were observed in October 2020 and May 2021 [17].

### Design

An ad-hoc and voluntary survey consisting of 5 multi-response questions was conducted using the Quizizz web application. Access to the questionnaire was provided through a numeric code, and respondents were allowed to remain completely anonymous by only providing an alias.

The survey included questions related to pandemic management both at the organizational and individual levels. The first three questions aimed to explore the changes that were introduced in the organization, workforce, and equipment. The remaining two questions were focused on individual coping strategies, one related to emotions and the other to problem-solving.

The research team designed the questionnaire based on information obtained from a prior study, in which six focus groups comprised of a total of 37 health professionals were asked: “What changes have occurred in your centers as a result of the COVID-19 pandemic?”, “What have you done that has worked well for you to feel better and face the care of COVID-19 patients in times of greatest uncertainty and crisis?”, and “What have you learned from other co-workers that works best to cope with the care of COVID-19 patients in times of greatest uncertainty and crisis?”. From this qualitative research technique, various ideas were gathered and categorized. Each of the survey questions was then formulated based on these categories. The response options consisted of the most commonly recurring ideas that emerged from the six focus groups. The readability of the content of the questions and the suitability of the expressions for participants from Argentina were analyzed.

### Characteristics of participants

Executives, including middle managers, and staff of health institutions in the province of Mendoza, directly involved in the response to the COVID-19 pandemic, who attended a reflective-formative session on patient safety and the impact of the COVID-19 pandemic and strategies for addressing the aftermath. This session took place on March 29, 2022, and all managers of public health institutions in the province of Mendoza were invited to attend (Table 1). The participants were informed about the purpose of the survey, and how the aggregate results would be used to foster dialogue during the session and draw conclusions. Informed consent was assumed by accessing the online questionnaire.

**Table 1:** Socio-demographic data of the people who attended the reflective-formative session on patient safety and the impact of the COVID-19 pandemic, held in Mendoza on March 29, 2022

Variables		N (%)
Sex		
	Man	32 (23.9)
	Woman	102 (76.1)
Age		
	Mean	48.4
	SD	9.7
Profession		
	Doctor	72 (53.7)
	Nurse	46 (34.3)
	General Services	16 (12.0)
Workplace		
	Hospital	78 (58.2)
	Core Services	25 (18.7)
	Primary Care	31 (23.1)

### Statistical analysis

The study was designed to identify the policy options that were most commonly utilized during the most critical moments of the COVID-19 pandemic. To achieve this, there were no limitations on the number of options to choose from, in order to avoid certain options being discarded for choosing others. Given the nature of the study, descriptive statistics were employed, specifically frequency analysis, to obtain information for each question.

## Results

Out of the 134 attendees to the reflective-formative session, a total of 60 people from hospitals and health centers accessed the survey. Of these, 45 (75.0%) individuals answered all of the questions (Table 2).

**Table 2:** Response rate per question

Question	N		N (%)	
1. What changes have occurred in the organization of the center that have been positive and that would not have been applied if it were not for the COVID-19 pandemic?	51		85	
2. What changes have occurred in the staff of the center that have been positive and that would not have been applied if it were not for the COVID-19 pandemic?	52		86.7	
3. What changes have occurred in the management of resources and equipment of the center that have been positive and that would not have been applied if it were not for the COVID-19 pandemic?	51		85	
4. Of the following emotion-focused strategies, which do you think helped you feel better and cope with caring for COVID-19 patients in times of greatest uncertainty and crisis? That is, both during your working day and to return the next day.	51		85	
5. Of the following problem-focused strategies, which do you think helped you feel better and cope with caring for COVID-19 patients in times of greatest uncertainty and crisis? That is, both during your working day and to return the next day.	45		75	

### Changes in institutions

At the organizational level, the participants agreed that the most positive changes observed were a clear increase in multidisciplinarity when performing tasks, and the incorporation of new protocols that have continued and led to improvements in the care received by patients. At the workforce level, there were restructurings in shifts, more specific training of personnel, and an increase in the number of workers who had improved conditions. Regarding resource and equipment management, needs were prioritized according to risks, and digital tools were enhanced (Table 3).

**Table 3:** Participants’ answers (N = 60). The option with the highest percentage of votes is highlighted in bold

Question	N	%	%*
**1. What changes have occurred in the organization of the center that have been positive and that would not have been applied if it were not for the COVID-19 pandemic?**			
Increased presence of clinical leadership	11	18.3	21.6
Telemedicine	20	33.3	39.2
**New protocols**	**38**	**63.3**	**74.5**
Multidisciplinary work	35	58.3	68.6
Without trying	9	15.0	-
**2. What changes have occurred in the staff of the center that have been positive and that would not have been applied if it were not for the COVID-19 pandemic?**			
Professional Support Programs	11	18.3	21.2
More specific training	31	51.7	59.6
Increase in the number of workers	30	50.0	57.7
**Shift restructuring**	**32**	**53.3**	**61.5**
Without trying	8	13.3	-
**3. What changes have occurred in the management of resources and equipment of the center that have been positive and that would not have been applied if it were not for the COVID-19 pandemic?**			
Centralization of resources	13	21.7	25.5
**Prioritize the need based on the risk**	**41**	**68.3**	**80.4**
Resources innovation	22	36.7	43.1
Empowerment of digital tools	34	56.7	66.7
Without trying	9	15.0	-
**4. Of the following emotion-focused strategies, which do you think helped you feel better and cope with caring for COVID-19 patients in times of greatest uncertainty and crisis? That is, both during your working day and to return the next day.**			
Religiosity	12	20.0	23.5
Meditation / Relaxation / Psychological Therapy	15	25.0	29.4
**Support from family or friends**	**45**	**75.0**	**88.2**
Escape (reading, sports, music, movies…)	27	45.0	52.9
Something I never dared to do before	8	13.3	15.7
Without trying	9	15.0	-
**5. Of the following problem-focused strategies, which do you think helped you feel better and cope with caring for COVID-19 patients in times of greatest uncertainty and crisis? That is, both during your working day and to return the next day.**			
Verify official information	20	33.3	44.4
Rest before starting work	16	26.7	35.6
**Focus on the positive of what was being done**	**33**	**55.0**	**73.3**
Team spirit	30	50.0	66.7
Feedback between colleagues	29	48.3	64.4
Without trying	15	25.0	-

* Percentage over the number of answers registered for each question

### Individual strategies

The most effective strategies to face the impact caused by the COVID-19 pandemic at the individual level were seeking social support through contact with family or friends, finding solace in hobbies such as music or sports, focusing on the positive aspects of what was being done, embracing team spirit, and exchanging feedback with colleagues (Table 3).

## Discussion

The responses originated during the COVID-19 pandemic represent an opportunity to identify changes and strategies that may prove effective in future similar situations. In addition, it is important to note that these proposals can serve as points to consider for strengthening the health system’s resilience in the post-crisis phase.

Although neither the protocols nor the multidisciplinary work are exclusively characteristic of crisis situations, their availability was essential to get ahead during the first wave of the pandemic, when uncertainty was greater. Therefore, it is important not to hastily return to the old normality and lose these advantages that could help delay the onset of fatigue due to repetitive tasks and, thus, improve the well-being of healthcare workers. Other research shows that moderate to high levels of exhaustion and low well-being among healthcare personnel are associated with poorer patient safety and an increase in clinical errors [18, 19].

Flexibility in workforces and adaptability to changing needs have also been essential. Under normal conditions, such changes are difficult to achieve [20], but during the pandemic, changes, adjustments, and rapid new incorporations had to be made due to the urgency of the needs and challenges that had to be addressed [21]. In this way, it is stressed that greater flexibility is needed to continue adapting to the changing needs of patients and healthcare professionals, also in the post-crisis phase [22].

Strategies focused on emotional management, especially support from family or friends, were more widely used than other strategies such as meditation or relaxation. In this sense, the barrage of bad news that characterized the first wave of the pandemic had a detrimental effect on the morale of healthcare workers who were required to return to work under those adverse conditions [23]. Many individuals chose to avoid the news or seek positive news as a means of personal protection for one’s emotional response to the pandemic. The constant bombardment of bad news was a stress factor that must be considered among the internal communication strategies in healthcare centers during a crisis [4]. An important lesson to be drawn for future emergencies is that bosses who shared positive news helped their teams. This characteristic reinforces the findings of other studies that have highlighted the importance of positive leadership [24–26]. Also, within individual strategies, it is important to encourage team spirit and provide feedback among colleagues to reinforce workers’ resilience [27].

Other studies examining measures to prevent the onset of burnout [28] have suggested providing employees with time to recover from stressful events, clearly defining the roles and expectations of the organization’s management, identifying appropriate rewards to recognize achievements, offering opportunities for teaching or mentoring students, promoting participation in professional organizations, ensuring transparency in decision-making, aligning personal expectations with organizational goals, and evaluating and adjusting work responsibilities with personal and professional expectations. Some of the measures implemented in the centers of Mendoza align with these recommendations, for example, restructuring the staff to allow rest periods, as well as the positive impact of strong clinical leadership.

Among all the initial proposals, this study points out that the incorporation of new protocols and the prioritization of needs based on risks had the most positive impact on healthcare workers. Likewise, the support of family and friends, along with a focus on the positive aspects of what was being done, helped to overcome the worst moments at the individual level.

We must learn from these experiences and use the recommendations highlighted in this study in the outbreak of future pandemics or health crises. In turn, this situation has revealed pre-existing problems that the pandemic brought to light. Specifically, issues related to the well-being of healthcare professionals, whose welfare is a fundamental element for the effective functioning of these health systems. This realization underscores the need to prioritize the physical, mental, and emotional health of healthcare workers. Therefore, addressing these issues could result in a higher quality National Health System, providing benefits to both healthcare providers and patients alike.

The need to build the resilience of health systems to cope with critical situations has been widely recognized in the aftermath of the COVID-19 pandemic [29]. Our study emphasizes the importance of leadership, teamwork, and team spirit, multidisciplinary, peer support and feedback, stress management strategies (relaxation, meditation) and psychological support in coping with challenging situations. These results are consistent with meta-analyses demonstrating the effectiveness of resilience interventions based on mindfulness, physical activity, psychoeducation, social support, cognitive skills, emotional regulation and relaxation [30, 31]. These elements together can be highly valuable in the development of an action protocol aimed at protecting the well-being of healthcare professionals in the face of future pandemics or health crises.

### Limitations

The data presented in this study is limited to the experience in Mendoza. When generalizing the data to other parts of the country or other countries, it must be considered the particular situation of each area.

The study’s design and priority given to maintaining user anonymity, given the complexity of the subject, rendered it impossible to carry out comparative analyses based on sociodemographic variables. Nevertheless, due to our focus on a specific group of individuals, we could not jeopardize their identity.

Another limitation is the potential for social desirability bias in self-reported data. Participants may have responded in a manner that they believed was socially acceptable or desirable, rather than providing honest answers, due to a desire to present themselves in a positive light.

It is also essential to consider the variability in the intensity of COVID-19 waves by territory, which may have differentially affected the coping and management strategies employed by professionals.

## Conclusions

The implementation of new protocols, shift restructuring, and prioritization based on risk have emerged as key measures implemented by healthcare organizations to cope with the initial waves of the pandemic. Similarly, individuals have highlighted the support of family and friends, as well as maintaining the focus on the positives of their work, as key factors to enhance resilience during the crisis. These changes must be considered in the event of future crises as elements that strengthen the resilience of healthcare professionals and teams.

## Data Availability

All data generated or analyzed during this study are included in this published article.

## Declarations

### Authors’ information

International collaboration within the framework of a joint project aimed at improving patient safety. Multidisciplinary team that strengthens the combination of perspectives from social and health disciplines.
